# Fournier’s gangrene: Causes, presentation and survival of sixty-five patients

**DOI:** 10.12669/pjms.323.9798

**Published:** 2016

**Authors:** Kerem Taken, Mehmet Resit Oncu, Muslum Ergun, Recep Eryilmaz, Canser Yilmaz Demir, Murat Demir, Mustafa Gunes

**Affiliations:** 1 Kerem Taken, Department of Urology, Medical Faculty, Yuzuncu Yil University, Van, Turkey; 2 Mehmet Resit Oncu, Department of Emergency, Medical Faculty, Yuzuncu Yil University, Van, Turkey; 3 Muslum Ergun, Department of Urology, State Hospital, Mus, Turkey; 4 Recep Eryilmaz, Department of Urology, State Hospital, Bitlis, Turkey; 5 Canser Yilmaz Demir, Department of Plastic Surgery, Medical Faculty, Yuzuncu Yil University, Van, Turkey; 6 Murat Demir, Department of Urology, Medical Faculty, Yuzuncu Yil University, Van, Turkey; 7 Mustafa Gunes, Department of Urology, Medical Faculty, Yuzuncu Yil University, Van, Turkey

**Keywords:** Dedridement, Fournier’s gangrene, Low socio-economic status, Predisposing factor

## Abstract

**Objective:**

To report our experience with Fournier’s Gangrene (FG) over the past eight years and evaluate the predisposing factors which affect the mortality.

**Methods:**

Sixty-five patients who were admitted to emergency surgical unit of our institution presenting with FG between January 2006 and August 2014 were included. The anatomical site of infective gangrene, predisposing factors, etiological factors, and outcomes were retrospectively reviewed.

**Results:**

Our cases included 8 women and 57 men. The average age of men was 51±13.9 (range 19-75) and the average age of women was 63±10.5 (range 52-76). Average hospitalization time was 9.2±6.6 days (range 5-25) days. The most frequent comorbid disease was diabetes mellitus and the most frequent etiology was perianal abscess. Colostomy was performed in 11 patients, orchidectomy in two patients, cystostomy in two patients. Notably, all of the 8 (12.3%) patients who died from FG had diabetes and low socioeconomic status. A total of six patients who died required more than one surgical debridement.

**Conclusions:**

Fournier’s gangrene is a severe surgical emergency, with a high mortality rate. Low socioeconomic status, diabetes and more than one debridement play a major role in mortality and morbidity.

## INTRODUCTION

Fournier’s gangrene (FG) is a fulminant necrotizing infection of the perianal and periurethral tissues that can disseminate even at the subcutaneous tissue of the thigh or the abdomen following the planes of the dartos fascia of the scrotum and penis, Colle’s fascia and Scarpa’s fascia.^1^

Predisposing factors include advanced age, primary anorectal/genitourinary infections and abscess, low socio-economic status, neurologic deficiency, diabetes mellitus, local trauma, urine leakage, recent perirectal or perineal surgery, periuretral/anal infection, alcohol abuse, immunosuppression.^2^-^4^ Patients with poor general health status are particularly prone to FG. This includes malnutrition or obesity, chronic renal failure, chronic liver disease, malignancies and other conditions causing immunosuppression.^5^,^6^ Infectious cases originating from the genitalia, the infecting bacteria probably pass through Buck’s fascia of the penis and spread along the dartos fascia of the scrotum and penis, Colles’ fascia of the perineum, and Scarpa’s fascia of the anterior abdominal wall. Wound cultures generally yield multiple organisms, implicating anaerobic-aerobic synergy.^7^

In the present study, we analyzed patients admitted to our institution’s emergency surgical unit presenting with FG in terms of the location of infective gangrene, predisposing factors, etiological agents and outcomes.

## METHODS

Hospital files of 65 patients diagnosed with FG between January 2006 and August 2014 were retrospectively reviewed. The review of the medical records of those patients included age, gender, and etiology, predisposing factors, duration between the onset of symptoms and first debridement, number of surgical interventions, culture findings, duration of hospital stay and clinical outcome. The 90-day mortality rate was calculated. The diagnosis of FG depended on the clinical symptoms/signs like erythema, rash, swelling, crepitus and necrosis in the perineal, perianal or genital areas. Culture samples were taken with swab margin of healthy tissue and wound. Once FG had been diagnosed by physical examination, all patients were treated with parenteral broad-spectrum triple antimicrobial agents, using penicillin G, an amino glycoside and metronidazole and received hemodynamic support when required. All patients underwent extensive debridement under spinal or general anaesthesia. The necrotic areas were debrided and the necrotic tissues were removed in several stages so that the bleeding tissues were observable. This procedure was done every other day. Then the wounds were cleansed with betadine, normal saline, and 2% oxygenated water and covered with nitrofurazon dressing. Tissue cultures were obtained routinely at time of debridement to identify the causative microorganism. Cystostomy was performed in cases with urethral or penile pathology. Colostomy was performed in cases with severe fecal contamination.

### Statistical Analysis

The data were analyzed by using the package program “SPSS for Windows 16.0” (SPSS Inc., Chicago, IL, USA). The risk factors that are thought to be able to influence the demographic characteristics and prognosis of the disease were compared according to the survival state. The comparisons were performed with *t*-test and the Mann-Whitney U test for continuous variables. The statistical significance was defined *p* < 0.05.

## RESULTS

A total of 65 patients were identified with FG. Eight of them were females (12.3%) and 57 were males (87.7%). The average age of men was 51±13.9 (range, 19-77). The average age of women was 63±10.5 (range, 52-76). Statistically, significant difference was found between the age and gender (*p* < 0.05). The mean duration from the onset of symptoms to admission to the hospital was 3.74±2.09 days (range: 1-10 days). Furthermore, the mean hospitalization time was 9.2±6.6 days (range: 5-25 days). Sepsis developed in 13 (20%) patients. The mean number of debridements was 2.5. A total of 12 (18.4%) patients required more than surgical debridement and 6 of them died. The age of 8 (12.3%) patients who died was 57.83±6.7, where the mean age of survivors was 51.67±14.9 and statistically, it was not found significantly (*p* = 0.34). Average hospital stay for survivors and the dead was not found significantly different (10.9±4.7 vs 7±1.7, *p* = 0.06). Most common etiological factors were anorectal conditions, urogenital disorders and trauma. No etiologic factors for FG were found in 13 (20%) patients and they were classified as idiopathic FG. Etiology of FG and predisposing factors in our patients are shown in Table-I. Incidence of diabetes in our study was 44.6%. Notably, all of the patients who died from FG had diabetes.

**Table-I:** Etiology of FG and predisposing factors in patients.

*Etiological factors*	*DM*	*MALG*	*CARD*	*COPD*	*UD*	*Total (n:)*
Perianal abscess	8	3	1	1	3	16
Perineal soft tissue infection	2		1	1		4
Ischiorectal abscess	3	1		1	2	7
Posthemorrhoidectomy	1				1	2
Fistula to the rectum	1	1		1		3
Sinuses to the skin					1	1
Prostatik biopsy	1				1	2
Urethral stricture	1		1		1	3
Perineal trauma	3	2	1		3	9
Scrotal abscess	4	1				5
Idiopatic		1	3	8	1	13
Total (n:)	29	8	7	12	13	65

DM: Diabetes mellitus, MALG: Malignancy, CARD: Cardiac disorders, COPD: Chronic Obstructive pulmonary disease, UD: Undefined

Culture results were obtained in 36 patients, and the most frequent bacterial organisms cultured from the wounds were *Escherichia coli* (n:9, 25%), anaerobic *Streptococcus* species (n:4, 11.1%), *Staphylococcus aureus* (n:4, 11.1%), *Enterobacter* spp (n:2, 5.6%), *Bacteroides* (n:2, 5.6%), *Pseudomonas aeruginosa* (n:2, 5.6%), *Proteus* species (n:2, 5.6%), *Clostridia* species (n:2, 5.6%) and mixed microorganisms (*Escherichia coli, Staphylococcus aureus*, anaerobs, n:9, 25%).

Multiple resection procedures were carried out in 52 (80%) cases. Colostomy was performed in 11 cases, diverting cystostomy in two. In addition to scrotal skin resection, unilateral orchidectomy in one and bilateral orchidectomy in another case were needed. Reconstructive surgery in 11 cases after the FG treatment was performed by urologists, all of the remaining were consulted to the plastic surgery for the reconstructive surgery. Vacuum assisted closure was not used in any patients.

## DISCUSSION

FG is characterized by rapid progression of infection in soft tissue caused by the synergistic action of several agents that extend along fascial planes. This necrosis is secondary to thrombosis of small vessels, which is due to endarteritis obliterans caused by the spread of microorganisms into the subcutaneous space that in addition to generating local edema, hypoxia, decrease in local blood supply, which helps anaerobic bacterial overgrowth. These microorganisms produce hydrogen and nitrogen that accumulate in tissues causing crepitation.^6^ The majority of the cases occur after 20 years of age.^6^,^8^,^9^ Studies reveal male predominance in FG where only minority of females affected when compared.^2^,^10^ In our study, the gender of patients were similar to literature with the 87.7 % male rate. Few reports are present in the literature with pediatric cases of FG.^11^ We also did not have any child patient.

The management options include urgent surgery, supportive and antibiotic therapy and hyperbaric oxygen.^12^ After initial radical debridement, open wounds are generally managed with sterile dressings or negative-pressure wound therapy.^13^ In a retrospective review, it was compared the efficacy of wound management with daily povidone iodine dressing versus Dakin’s solution (sodium hypochlorite) which has wide antimicrobial efficacy against aerobic and anaerobic organisms. The authors found that the length of hospitalization was significantly shorter in patients managed with Dakin’s solution compared with iodine dressing.^14^ Also, it was reported the therapeutic effects of honey in Fournier’s gangrene as an adjuvant therapy because of its ability to inhibit microbial growth likely related to the osmotic effect of its high sugar content.^15^ In our study the management of FG was underscored by four main principles: rapid and aggressive surgical debridement of necrotized tissue, hemodynamic support with urgent resuscitation with fluids, broad-spectrum parental antibiotics and managed with nitrofurazon dressing.

The mortality of this entity remains high, despite the advances in medical care facilities for FG patients, the mortality rate reported in the literature ranging from 4% to 80%.^2^,^6^,^8^,^16^ Likewise, the mortality rate in our study was 12.3%. It has been reported that mortality risk was increased in patients who required more than one surgical debridement.^16^ In our series, six patients of total 8 patients who died required more than one surgical debridement. We are of the same opinion that more than one surgical debridements is related with mortality.

DM is the most common predisposing factor with an incidence of 46-76.9%.^3^ Similarly, 44.6% of all patients in our study had DM, and 27.6% of these patients died. It is also worth noting that all patients who died had DM. On the other hand, some authors reported that DM was not associated with mortality and morbidity.^17^ In our study, 44.6% of all patients had DM, and 27.6% of these patients died. It is also worth noting that all patients who died had DM. Hence DM is a major predisposing and prognostic factor according to our results.

Early hospitalization plays an important role in survival which is related with the patient’s socio-economic status. As some authors have stated, low socio-economic status as in our area remains an important role in the late admittance of the patient. As a result, longer duration of hospital stay, higher numbers of the debridement which leads to morbidity and mortality.^18^,^19^ Low socio-economic status (especially patients from rural areas) was detected in the majority of our patients. Also, it was observed that all patients who died had low socio-economic status. The gap between the onset of symptoms and admission to the hospital for these patients, the number of the debridements, the mortality and morbidity rates were higher than their urban counterparts.

In our study, anorectal diseases were the leading cause (n:33, 50.8%), followed by idiopathic 13 (20%) and others (trauma, scrotal abscess, uretral stricture and prostate biopsy) in 6 (10%) in consistency with literature.^20^,^21^ It is also worth noting that FG developed in two patients subsequent to prostate biopsy after transrectal ultrasound guided prostate biopsy. One patient had diabetes while the other did not have any predisposing factor. Time interval between intervention and hospital admittance was 7 days in diabetic patient and 10 days in the latter one. Both patient did not receive prophylactic antibiotic. Therefore, our recommendation is to use routine antibiotic prophylaxis before prostate biopsy. Conventional radiology can be helpful in assessing some cases revealing the presence of gas in soft tissues. In our series, no methods were used for imaging studies and all diagnosis were made on clinical basis.

Cultures from the wounds commonly show polymicrobial infections by aerobes and anaerobes, which include coliforms, klebsiella, streptococci, staphylococci, clostridia, bacteroids, and corynbacteria.^12^ *E coli* has been reported to be the most common organism isolated from the wound cultures (43-80%). Anaerobes are less frequently isolated than expected, which could be because of technical faults.^22^,^23^ Rare reports of other organisms being cultures include *Candida albicans* and *Lactobacillus gasseri*.^12^,^24^,^25^ In our study, *E. coli* has been identified as the major microbial factor.

Colostomy is sometimes needed to decrease fecal contamination.^26^,^27^ Adequate urinary diversion can usually be accomplished by a foley’s catheter unless urethral disruption. In such situations, cystostomy is needed.^27^ Colostomy was needed for eleven of our patients and suprapubic cystocatheter was needed in two patients. In a retrospective review of 29 patients over a 13-year period, Baskin et al reported that only three patients (all more than 65 years of age) underwent orchidectomy.^17^ In a recent study by Ayan et al, who retrospectively reviewed records of 41 patients, bilateral orchidectomy were done in four (9.7%) patients and unilateral orchidectomy in five (12.1%) patients due to necrosis.^28^ In our study, two patients had orchidectomy because of gangrenous testis. A patient in whom colostomy, cystostomy and orchidectomy was performed died. So that more than one surgical intervention can play a role in mortality.

## CONCLUSIONS

Fournier’s gangrene continues to be a severe surgical emergency. Low socioeconomic status, diabetes and more than one debridement play a major role in morbidity and mortality. Antibiotics and aggressive debridement have been broadly accepted as the standard treatment, even so the death rate remains high.
